# Predicting the wicking rate of nitrocellulose membranes from recipe data: a case study using ANN at a membrane manufacturing in South Korea

**DOI:** 10.1007/s44211-024-00540-8

**Published:** 2024-04-10

**Authors:** Janith Dissanayake, Sung Bong Kang, Jihoon Park, Fang Yinbao, Sungryul Park, Min-Ho Lee

**Affiliations:** 1 Newnop Co. Ltd, 2209, 22nd Floor, Building A, 58-1, Giheung-Ro, Giheung-Gu, Yongin-Si, Gyeonggi-Do South Korea; 2 Department of Civil and Environmental Engineering, Seoul National University, 1 Gwanak-Ro, Gwanak-Gu, Seoul, 08826 South Korea; 3 Department of Medical Device Industry, Yonsei University College of Medicine, 50 Yonsei-Ro, Seodaemun-Gu, Seoul, 03722 Republic of Korea; 4 UMTR Co.,Ltd., 424-ho (Center M) 33, Sagimakgol-ro 62beon-gil, Jungwon-gu, Seongnam-si, Gyeonggi-Do Republic of Korea; 5 School of Integrative Engineering, 84 Heukseok-Ro, Dongjak-Gu, Seoul, 06974 Republic of Korea

**Keywords:** Deep Learning, Nitrocellulose membranes, Lateral flow assays, Artificial neural networks, Wicking rate

## Abstract

Lateral flow assays have been widely used for detecting coronavirus disease 2019 (COVID-19). A lateral flow assay consists of a Nitrocellulose (NC) membrane, which must have a specific lateral flow rate for the proteins to react. The wicking rate is conventionally used as a method to assess the lateral flow in membranes. We used multiple regression and artificial neural networks (ANN) to predict the wicking rate of NC membranes based on membrane recipe data. The developed ANN predicted the wicking rate with a mean square error of 0.059, whereas the multiple regression had a square error of 0.503. This research also highlighted the significant impact of the water content on the wicking rate through images obtained from scanning electron microscopy. The findings of this research can cut down the research and development costs of novel NC membranes with a specific wicking rate significantly, as the algorithm can predict the wicking rate based on the membrane recipe.

**Graphical abstract:**

Correlation matrix displaying the distribution of the variables and linear correlations

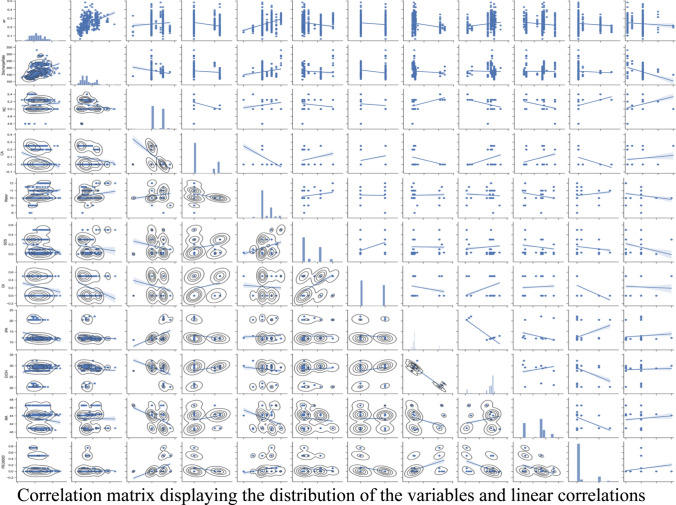

## Introduction

**Fig. 1:**
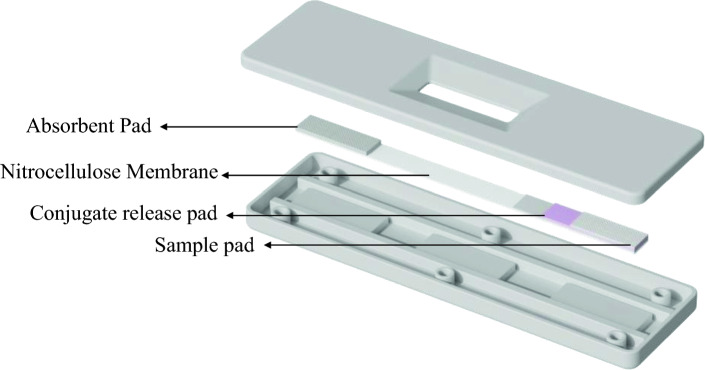
Breakdown of the components of a lateral flow assay (LFA). The image was reproduced with permission from the UMTR Co., Ltd, 2023

Coronavirus disease 2019 (COVID-19), caused by the SARS-CoV-2 virus, has spread across more than 200 countries, and infected more than 660 million people, taking the lives of more than 6.7 million people worldwide [1]. Despite these challenges, researchers worldwide have managed to create more than 400 diagnostic tests and collection kits, including 63 home collection, 32 pooling, 55 point-of-care, 19 multi-analyte, and 13 at-home products that have been approved for emergency use by the US Food and Drug Administration [2]. Ritchie et al. [3] reported that over 450 million tests had been performed in the USA as of May 2021. This emphasizes the importance of the research and development of reliable and speedy diagnostic kits to ensure preparedness for pandemics of large magnitude [4]. A lateral flow assay (LFA), most known as a rapid test, has been widely utilized more over polymerase chain reaction (PCR) tests due to its ease of use, rapidness, and cost effectiveness [5, 6]. In fact, Biby et. al [4]. reported that 44 LFAs were authorized by the FDA in the USA for COVID-19 in vitro diagnostics. Simply, an LFA can be described as a system consisting of an analytical membrane that act as an autarkic microfluidic pump system capable of transporting the pretreated (if necessary) sample from the sample pad to conjugate pad [7]. While diffusing through the conjugate pad, the sample is mixed with gold nanoparticles (AuNPs), biomolecule conjugates, and additives, which react with the antibodies on the membrane [4, 7] (Fig. 1). The membrane made of Nitrocellulose (NC) plays a critical role as all the biological reactions leading to the signal generation taking place on the Nitrocellulose membrane (NCM). The performance of an NCM as an analytical membrane depends on the thickness of the NC membrane and the pore size. The layer thickness of the NC membrane affects the sample volume needed for the test. Mansfield, 2009 [8] reported that the ideal layer thickness should be within the range of 100–150 µm. The pore size of the membrane governs the capillary flow rate, which determines the sensitivity and readout times. Although a high flow rate will provide a better readout time, it can also decrease the time available for AuNPs to react or bind with the targeted antibodies [9]. Despite much research and development, optimizing and designing the NCMs to reach a particular capillary flow rate remains a challenge.

The wicking rate of a membrane refers to the surface-driven imbibition process due to capillary suction. Gasperino et al., Fries & Quere, and Masoodi et al. [10–12] discussed the application of Darcy’s law and the Richards equation, whereby the wicking rate was determined by the volume averaging of certain properties: porosity, permeability, fiber radius, and pore radius [13, 14]. Lucas and Washburn derived a solution for porous mediums, considering the capillary pressure developed in a cylindrical capillary with the assumption that the porous medium is a collection of cylindrical capillaries [15]. Altschuh et al. [7] demonstrated that the estimated effective pore radius is approximately eight to ten times higher than the geometric means. They argued that use of solutions such as the Young–Laplace equation and Lucas and Washburn solutions are not applicable in the context of membranes, as the structures are often non-axisymmetric and closed. Similarly, much research has been conducted to evaluate the flow within the microstructures; however, the authors were not able to find any research that investigated the effect of the membrane recipe on the wicking rate.

NCMs are manufactured by mixing chemicals such as nitrocellulose, their solvent, and nonsolvent additives. The quantities of each compound and the membrane manufacturing process influence the pore size, contact angle, and porosity of the membrane, which, in turn, affect the wicking rate [16, 17]. In this paper, we have attempted to use an artificial neural network (ANN) to predict the membrane wicking rate from just the membrane recipe data and the machine control data.

## Materials and methods

### Materials

The raw data for this analysis were obtained from UMTR Co., Ltd (UMTR) located at 8, Suseong-ro, Gwonseon-gu, Suwon-si, Gyeonggi-do, 16426, Republic of Korea, . The company manufactures NCMs for Influenza-A LFAs. The chemicals used for NCM manufacture are Nitrocellulose ([C_6_H_9_(NO_2_)O_5_]*n*, purchased from KCNC 51, Wanjusandan 4-ro, Bondgong-eup, Wanju-gun, Jeollabuk-do, Korea), CA (cellulose acetate, [C_6_H_7_O_2_(OH)_3_]*n*, purchased from Samchun Chemical Co., Ltd. 117, Sandan-ro 16 beon-gil, Pyeongtaek-si, Gyeonggi-do, Republic of Korea), DI water (Samchun Chemical Co., Ltd. 117, Sandan-ro 16beon-gil, Pyeongtaek-si, Gyeonggi-do, Republic of Korea), SDS (sodium dodecyl sulfate, NaC12H25SO4, Samchun Chemical Co., Ltd. 117, Sandan-ro 16 beon-gil, Pyeongtaek-si, Gyeonggi-do, Republic of Korea), caster oil (caster oil, Samchun Chemical Co., Ltd. 117, Sandan-ro 16beon-gil, Pyeongtaek-si, Gyeonggi-do, Republic of Korea), IPA (2-propanol, C_3_H_8_O, Samchun Chemical Co., Ltd. 117, Sandan-ro 16beon-gil, Pyeongtaek-si, Gyeonggi-do, Republic of Korea), EtOH (ethyl alcohol, C_2_H_6_O, Samchun Chemical Co., Ltd. 117, Sandan-ro 16beon-gil, Pyeongtaek-si, Gyeonggi-do, Republic of Korea), MA (methyl acetate, C_3_H_6_O_2_, Samchun Chemical Co., Ltd. 117, Sandan-ro 16beon-gil, Pyeongtaek-si, Gyeonggi-do, Republic of Korea), PEG6000 (polyethylene glycol 6000, (2H_6_O_2_)*n*; H(OCH_2_CH_2_)*n*OH, purchased from Samchun Chemical Co., Ltd. 117, Sandan-ro 16beon-gil, Pyeongtaek-si, Gyeonggi-do, Republic of Korea), and TWEEN 20 (polyoxyethylene sorbitan monolaurate, C58H114O26, purchased from Samchun Chemical Co., Ltd. 117, Sandan-ro 16beon-gil, Pyeongtaek-si, Gyeonggi-do, Republic of Korea).

### Membrane manufacturing process

**Fig. 2:**
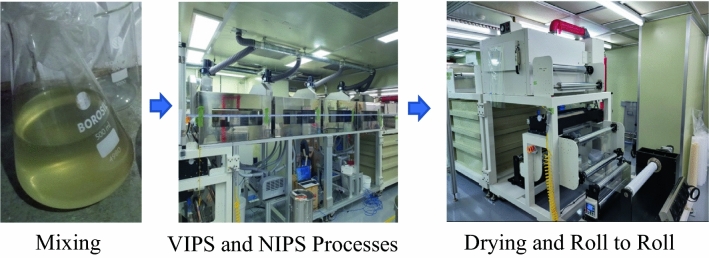
The Nitrocellulose membrane manufacturing process. The processes include mixing, vapor-induced phase separation (VIPS), nonsolvent-induced phase separation (NIPS), drying, and finally a roll-to-roll process

The Nitrocellulose (NC) was quantified based on the solid content followed by the addition of ethyl alcohol, 2-Propanol, DI water, sodium dodecyl sulfate, and polyethylene glycol. A complete solution was prepared by stirring with the NC for three hours using a three-horsepower stirrer. The residual materials were removed using a polyester filter. After filling the reaction tank, it was used after removing air bubbles. The membranes were fabricated using vapor-induced phase separation (VIPS) and nonsolvent-induced phase separation (NIPS) processes, sequentially (Fig. 2).

**Fig. 3:**
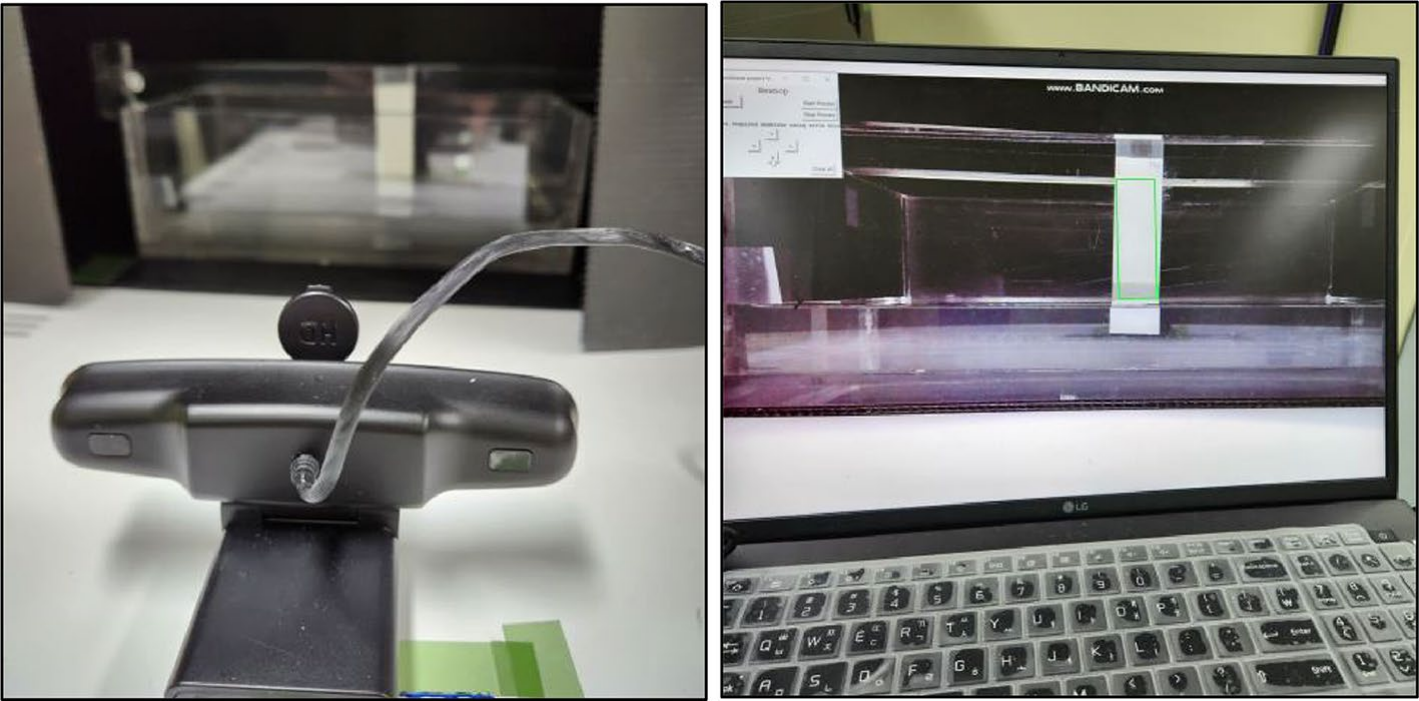
The machine vision system to measure the membrane wicking rate

The VIPS used water vapor to facilitate the required humidity conditions. The relative humidity was maintained at 80%, and the temperature was maintained at 25 °C. The VIPS tanks maintained 80% relative humidity, by continuously introducing water vapor via the exhaust holes installed at both ends of each chamber at a speed of 50 cc/min. This facilitated the removal of the accumulated solvent during the development of the web pass. This process facilitated the phase separation. During the NIPS process, the water temperature was maintained at 25 °C. An optical polyethylene terephthalate (PET) film purchased from SKC (Block B, The K Twin Towers, 50 Jongro-1-gil, Jongro-gu, Seoul, South Korea) was used during the process. For coating, a mono pump capable of quantitatively injecting the solution was used, and the coater used a slot die to control the coating gap. The web pass was designed to accurately match the progress speed by using a servo motor. The production line maintained a constant speed (0.1–1.0 m/min) throughout the VIPS and NIPS processes, drying furnaces, and final winding in the rewinder. The drying furnace was programed to increase the temperature from medium to high temperatures (a step increment control program was used to set the temperature to 70 °C, 90 °C, and 110 °C) to ensure that the 100 um nitrocellulose membrane was sufficiently dry. Scanning electron microscopy (SEM) (HITACHI FE SEM, SU8600, Tokyo, Japan) images of the manufactured membranes were taken to assess the pore diameters and pore structures. The wicking rate plots of the manufactured membranes were assessed digitally through a machine vision system (Fig. 3). Membranes of size 25 × 75 mm were clamped into a supporting device as shown in the Fig. 3, and one edge was inserted into a thin layer of distilled water. The time taken for the wetted distance to reach 40 mm was defined as the wicking time. In the experiment, it was assumed that the effects of swelling and evaporation are negligible.

### Method of analysis

All the data (5 years of research data with research and development cost of 10 million USD) were analyzed, and the ANN was developed using the TensorFlow (v2.12.0), Keras, Numpy(1.21.5), SciKit-Learn(1.1.1), and Pandas (1.4.4) libraries in Python 3.9. Firstly, the data (731 data points) were preprocessed to remove any missing values and outliers. Next, the preprocessed data (Table 1) were descriptively analyzed. Afterwards, the preprocessed data were then split into testing and training datasets with a split ratio of 20% with zero random state. The training dataset was normalized prior to fitting to the machine learning models and ANN. The initial ANN was built with an input layer with 12 input dimensions and 128 neurons and two hidden layers with 128 neurons with the Relu activation function. The final layer (output layer) consisted of 1 unit (neuron). The model was compiled with the mean square error loss function and the Adam optimizer function over 100 epochs. The model was evaluated with the predicted wicking rates from the test features. The model was then compared for the accuracy with the test labels. The model hyperparameters (optimizer, epochs, and batch size) were optimized to prevent overfitting.

**Table 1:** An extract of the preprocessed data that contained 557 records

Wicking Rate	Speed	Viscosity	Discharge Rate	Thickness	NC	CA	Water	SDS	Oil	IPA	EtOH	MA	PEG6000	Tween20
0.286	0.6	1145	230	106	5	0	10	0.03	0	11.536	28.84	44.41	0	0
0.362	0.6	1145	230	112	5	0	10	0.03	0	11.536	28.84	44.41	0	0
0.272	0.6	1119	230	106	5	0.2	10	0.01	0	11.536	28.84	44.41	0	0.1
0.258	0.6	1199	230	99	5	0.2	10	0.01	0	11.536	28.84	44.41	0	0.1
0.231	0.6	1199	230	101	5	0.2	10	0.01	0	11.536	28.84	44.41	0	0.1
0.183	0.6	1164	180	92	5.1	0.25	10	0.03	0	11.536	28.84	44.41	0	0.03
0.206	0.6	1164	180	86	5.1	0.25	10	0.03	0	11.536	28.84	44.41	0	0.03
0.194	0.6	1164	180	88	5.1	0.25	10	0.03	0	11.536	28.84	44.41	0	0.03
0.219	0.6	1049	230	96	5.1	0	10	0.03	0	11.536	28.84	44.41	0	0.03
0.258	0.6	1049	230	97	5.1	0	10	0.03	0	11.536	28.84	44.41	0	0.03
0.272	0.6	1004	230	104	5.1	0	10	0.03	0	11.536	28.84	44.41	0	0.03
0.272	0.6	1025	230	106	5	0	10	0.03	0	11.536	28.84	44.41	0	0.03
0.300	0.6	897	250	100	5	0	10	0.03	0	11.536	28.84	44.41	0	0.03
0.315	0.6	897	250	104	5	0	10	0.03	0	11.536	28.84	44.41	0	0.03
0.362	0.6	897	250	104	5	0	10	0.03	0	11.536	28.84	44.41	0	0.03
0.231	0.6	967	230	95	5	0	10	0.03	0	11.536	28.84	44.41	0	0.03
0.286	0.6	967	230	105	5	0	10	0.03	0	11.536	28.84	44.41	0	0.03
0.330	0.6	967	230	110	5	0	10	0.03	0	11.536	28.84	44.41	0	0.03
0.286	0.6	968	230	103	5	0	10	0.03	0	11.536	28.84	44.41	0	0.03
0.272	0.6	968	230	108	5	0	10	0.03	0	11.536	28.84	44.41	0	0.03
0.272	0.6	1034	230	110	5	0	10	0.03	0	11.536	28.84	44.41	0	0.03
0.244	0.6	1025	230	104	5	0.2	10	0.01	0	11.536	28.84	44.41	0	0.1
0.286	0.6	840	230	96	5	0.2	10	0.01	0	11.536	28.84	44.41	0	0.1
0.362	0.6	840	230	98	5	0.2	10	0.01	0	11.536	28.84	44.41	0	0.1
0.300	0.6	960	230	93	5	0	11	0.03	0	11.092	27.73	46.58	0	0.1

*The measurement units are as follows. Wicking rate- mm/sec, speed-from 0.3 m/min to 1.0m/min, viscosity-from 500 to 1500 cps, thickness-from 80 to 120 um

## Results and discussion

### Descriptive statistics and linear regression

**Fig. 4:**
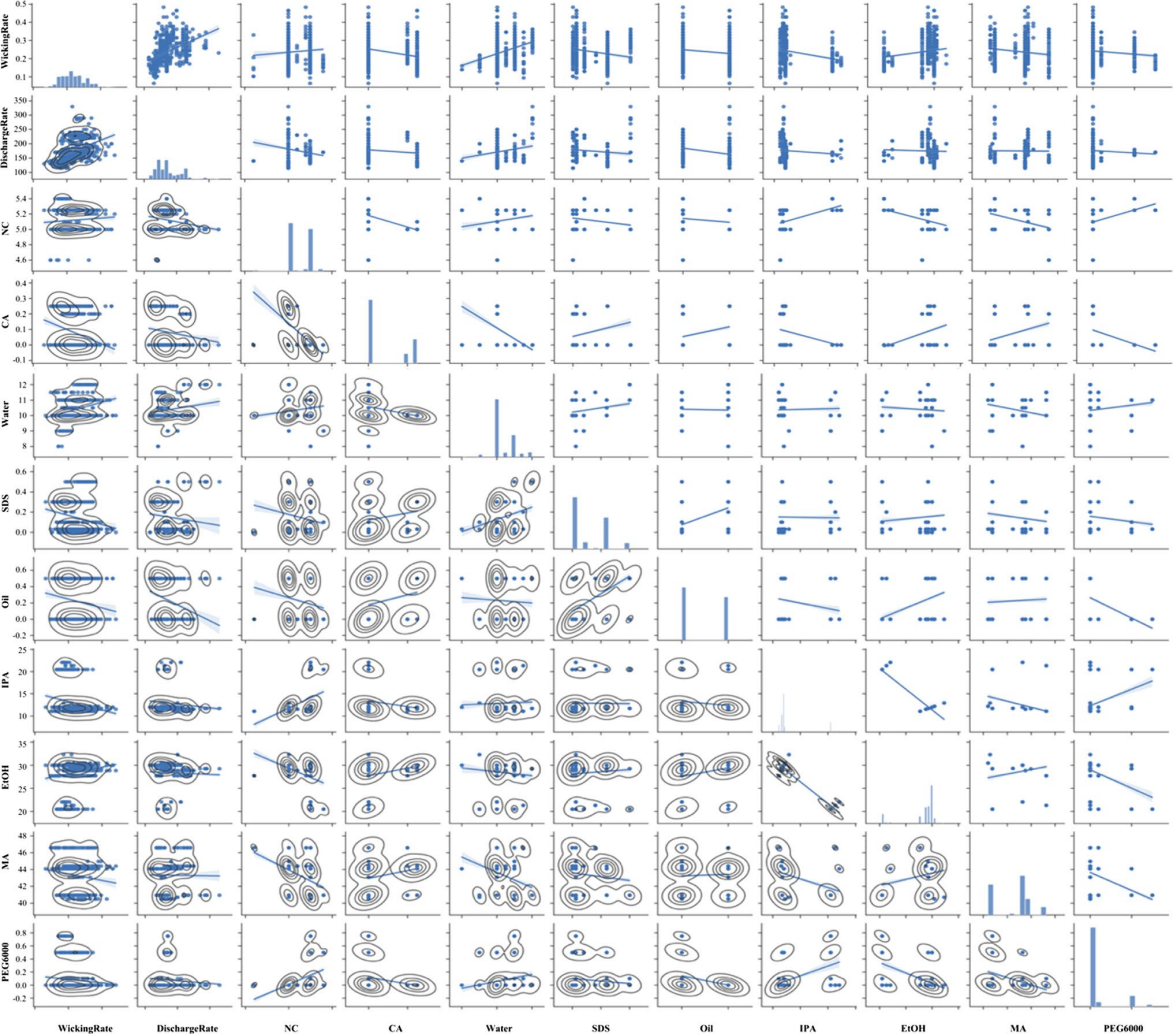
The correlation matrix displaying the distribution of the variables and linear correlation among the parameters

The preprocessing of the initial dataset reduced the data records to 557 from 731 records, due to missing values contained in the dataset. Several outliers were removed based on expert opinion and the results obtained for similar recipes and operating conditions. The summary statistics for the preprocessed data set are presented in Table 2. The wicking rate varied from 0.06 mm/s to 0.48 mm/s with a mean value of 0.24 mm/s. The thickness of the membranes ranged from 62 to 120 mm with a mean value of 96 mm. The discharge rate of the equipment varied from 115 m^3^/m to 330 m^3^/m. Low discharge rates were associated with thicker membranes. The thickness of the membrane was significantly affected by the discharge rate of the equipment (*p* < 0.05). The PEG600, Tween20, caster oil, CA & SDS chemicals were introduced to only few samples. Consequently, the composition of the components of the membrane recipe varied drastically. The amount of water, SDS, and MA added had a good distribution across a wider range, while the rest of the chemicals were concentrated on two weight composition points (Fig. 4). Therefore, the w/w % distributions for most of the chemicals were skewed to one side. Yacob [18] explained in his thesis that production engineering aims to reduce the variations in the components in the manufacturing process to reach higher efficiencies. Similarly, UMTR Co., Ltd. narrowed down the chemical composition ranges to optimize their processes, which may have contributed to the skewness observed in some distributions.

**Table 2:** The summary statistics (count, mean, standard deviation, minimum, maximum, and 25%, 50%, and 75% quartiles) for the data considered in this study

	Wicking rate (mm/sec)	Discharge rate (Unit)	Thickness (mm)	NC (w /w %)	CA (w /w %)	Water (w /w %)	SDS (w /w %)	Oil (w /w %)	IPA (w /w %)	EtOH (w /w %)	MA (w /w %)	PEG6000 (w /w %)	Tween20 (w /w %)
Count	557	557	557	557	557	557	557	557	557	557	557	557	557
Mean	0.239597	174.8743	95.87814	5.119838	0.081688	10.37702	0.149031	0.225314	12.87671	28.39904	43.3237	0.077379	0.086858
Std	0.069294	37.04438	10.46621	0.139425	0.113069	0.664931	0.155221	0.249002	2.943026	2.959135	1.81164	0.186914	0.040726
Min	0.064464	115	62.22222	4.6	0	8	0	0	11.092	20.475	40.4985	0	0
25%	0.182857	150	89	5	0	10	0.03	0	11.7	28.84	40.95	0	0.1
50%	0.231429	170	96	5	0	10	0.03	0	12	29.25	44.1	0	0.1
75%	0.285714	200	103.6667	5.25	0.2	11	0.3	0.5	12	30	44.41	0	0.1
Max	0.482857	330	120.5556	5.4	0.25	12	0.5	0.5	22.05	32.31	46.58	0.75	0.3

**Fig. 5:**
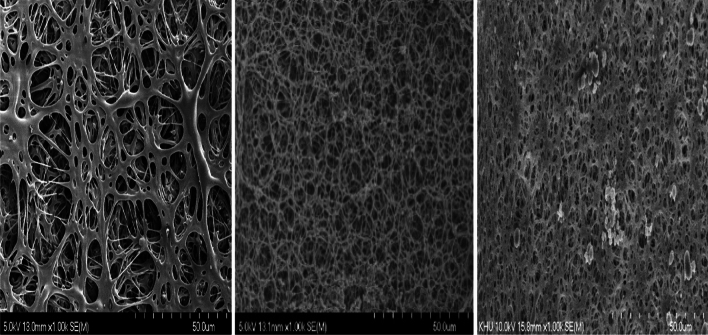
The SEM images for 8 (Left), 9 (Middle), and 10% (Right) water content while maintaining NC -5%, CA: MA 1:1,IPA:EtOH-1:2.5, SDS -0.5,TWEEN 20- 0.05

The wicking rate was significantly (*p* < 0.05) correlated with the water content, SDS, and EtOH composition. The chemical composition affected the thickness of the membranes and the diameter of the pores, which may have contributed to the above observation. These phenomena were further corroborated by the SEM images. A sample of SEM images is provided as Fig. 5, which highlights the increase in pore size when the water content changed from 8 to 10%. The larger pores promoted the lateral flow within the membrane which resulted in a higher wicking rate. The chemical composition of the casting solution changed the viscosity of the mixture (within the range 543–1233 mPa.s). The changes in viscosity affected the membrane morphology due to its ability to influence the solvent/non-solvent phase inversion rate [19]. Lower viscosities allow excessive solution to penetrate porous support material, that will reduce the pore diameter [20].

Aside from the chemical composition, the operating conditions of the manufacturing line also affected the wicking rate of the membrane. The wicking rate was positively correlated to the discharge rate. The SEM analysis showed that high discharge rates were attributable to the increased pore sizes. Although the temperature program and the humidity control during the manufacturing process can affect the wicking rate [21], this study did not investigate the effects of these on the wicking rate. Instead, we kept the temperature program and the humidity control measures constant throughout the entire research period, as described in the Materials and Methods section of this article. Ahmad et al. [10] in their study demonstrated that porosity of the membrane increased with the increasing drying temperature during the manufacturing process. They observed this phenomenon for a range of polymer content. Therefore, the authors believe that our experiments should be extended to various temperature profiles for a more comprehensive study. Besides, the external temperature may also affect the membrane morphology, as the mixture gets exposed to the external environment during the manufacturing process. In this study the external environment temperature was maintained at 25 °C using a HVAC system. Therefore, further analysis is needed to determine the effect of the external temperature on the membrane.

**Fig. 6:**
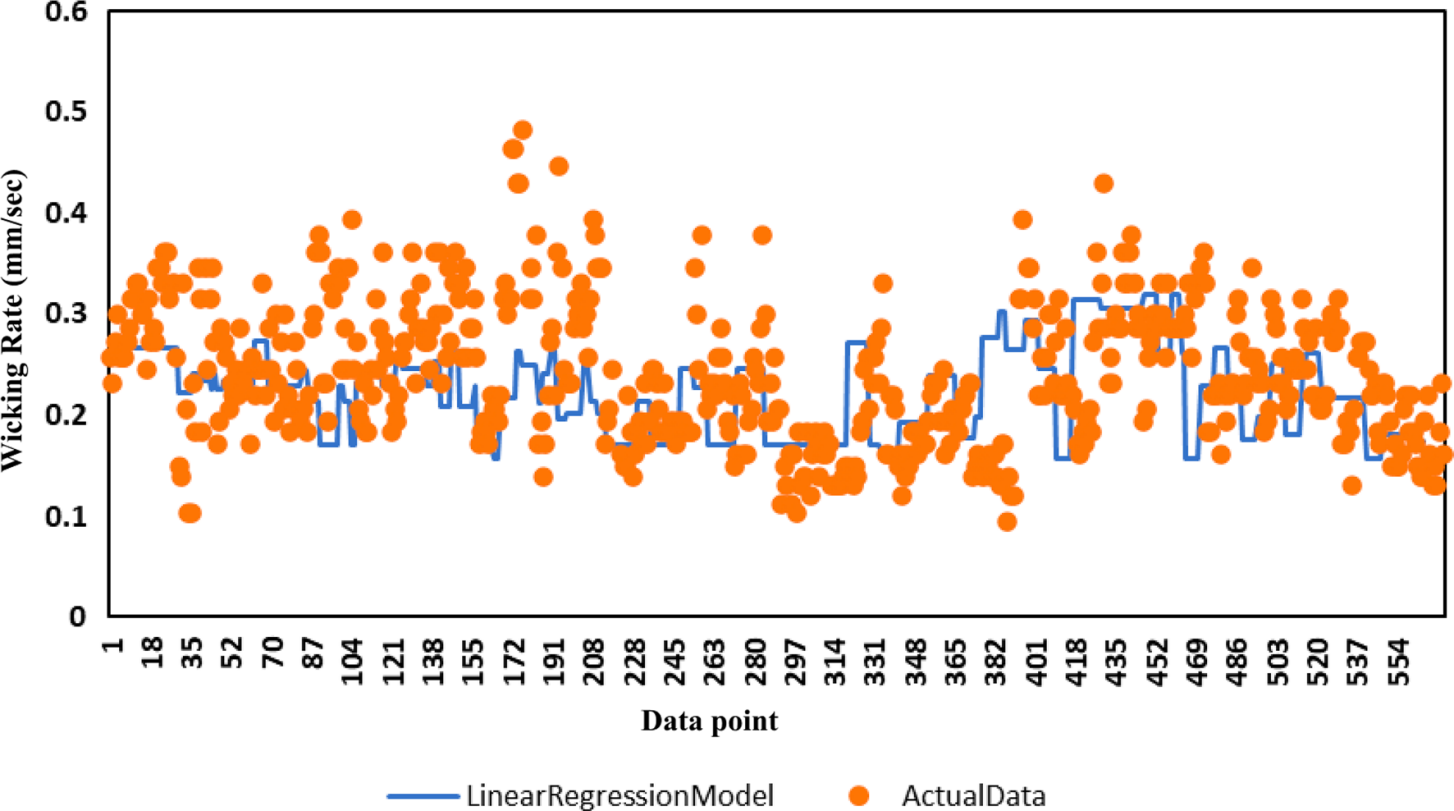
Multiple regression analysis of the dataset. The predicted wicking rate of the Linear regression model is presented by the blue line and the actual wicking rates for those 557 data points are presented in orange color markers

The multiple linear regression analysis (Fig. 6) performed with all the data had an R-square value of 0.503. The relatively low fit is attributable to the poor linear relationship that the majority of the parameters had with the wicking rate. The correlation coefficients for the discharge rate, thickness, NC, CA, water, SDS, oil, IPA, EtOH, MA, PEG6000, and Tween20 were -0.888, 0.002, 0.032, -0.024, 0.032, -0.076, 0.02, -0.006, 0.001, -0.008, -0.083, and -0.015, respectively. Most of the coefficients were less than 0.5, suggesting that the relationships were nonlinear except in the case of the discharge rate.

### Prediction using ANN

The predictions using the ANN model proved to be more accurate with a loss of 0.059. The loss function was carefully observed to avoid any over/underfitting. This was further prevented by optimizing the hyperparameters considered in this study. The ANNs use nonlinear algorithms to create relationships with nodes. That may be the reason why the ANN provided better results compared to the multiple linear regression. Our developed model was tested only with a portion of the original dataset that we built in collaboration with UMTR Co., Ltd. The model did not consider the impact of the temperature and the humidity conditions of the environments and the conditions of the NIPS and VIPS processes in this study, as we maintained them at a fixed state.

## Conclusion

This research explored the possibility of using ANNs to predict the membrane wicking rate using the membrane recipe generation data and parameters of the VIPS and NIPS processes. The company UMTR Co., Ltd had spent nearly 10 million USD to develop membranes of specific wicking rates to be used for Influenza A and Covid-19. Although the manufacturing equipment was available, it took significant trial and error process to identify the appropriate recipe that will generate a suitable membrane. The time consumed and cost of developing a fit-for-purpose membrane therefore has created a barrier for research and even manufacturing. In this study we demonstrated that the ANNs can accurately predict the membrane wicking rate, and this can assist researchers and membrane manufacturers in manufacturing membranes with a specific wicking rate more quickly. Besides, the authors observed that water content, SDS, and EtOH composition had a significant impact on the morphology of the membrane and the wicking behavior of the membrane. These findings will assist researchers in focusing on which parameters to optimize if the problem lies primarily with the wicking rate of the membrane. This is the first study to predict the wicking rate using the samples obtained from membrane manufacturers and there is a need to assess other similar materials to find out its feasibility. Furthermore, we recommend expanding the case studies by varying the temperature and humidity of membrane manufacturing process to make the research more comprehensive.

## Data Availability

The data may be made available upon request for noncommercial purposes.
